# Applications of Artificial Intelligence Technologies in Nursing for Noncommunicable Chronic Diseases: A Scoping Review

**DOI:** 10.1155/jonm/5288231

**Published:** 2026-08-26

**Authors:** Shuying Miao, Zheng Zhang, Chunxiang Bao

**Affiliations:** ^1^ Department of Nursing, The First Affiliated Hospital, Zhejiang University School of Medicine, Hangzhou 310003, China, zju.edu.cn

**Keywords:** artificial intelligence, chronic disease management, NCDS, nursing care, scoping review

## Abstract

**Background:**

Noncommunicable chronic diseases (NCDs) account for approximately 41 million deaths each year and 74% of global deaths. Artificial intelligence (AI) is increasingly used for risk prediction, monitoring, and decision support in chronic disease care, but its nursing‐specific applications, implementation maturity, and ethical reporting remain incompletely mapped.

**Aim:**

To map evidence on AI applications in nursing care for NCDs, with attention to application contexts, nursing roles, technical characteristics, care phases, outcomes, and ethical governance.

**Methods:**

A scoping review was conducted and reported in accordance with the Preferred Reporting Items for Systematic Reviews and Meta‐Analyses extension for Scoping Reviews (PRISMA‐ScR). MEDLINE, CINAHL, Web of Science, PsycINFO, IEEE Xplore, ACM Digital Library, and CNKI were searched from 1 January 1985 to 25 June 2025. Grey literature sources and reference lists were also searched. Data were charted using a structured extraction form and synthesised with descriptive statistics and content analysis.

**Results:**

Forty‐three studies published between 2005 and 2025 were included. Research increased after 2020 (*n* = 34, 79.07%), and China and the United States each contributed 11 studies (25.58%). Oncology nursing was the most common disease area (*n* = 17, 39.53%). The most frequently reported AI technologies were natural language processing (*n* = 14, 32.56%) and risk prediction models (*n* = 12, 27.91%). AI was mainly used for clinical decision support (*n* = 17, 39.53%) and risk assessment (*n* = 14, 32.56%), whereas applications supporting nursing intervention design and outcome evaluation were limited. Ten studies (23.26%) did not report ethics approval.

**Conclusions:**

AI applications in nursing care for NCDs are expanding, but current evidence remains concentrated in assessment‐oriented and decision‐support functions. Gaps persist in intervention implementation, outcome validation, data representativeness, and ethical oversight.

**Implications for Nursing Management:**

With AI currently concentrated in hospital‐based settings and assessment‐oriented functions, and with 32.56% of included studies not reporting nursing or patient outcome validation, nursing leaders should prioritise extending AI implementation and governance to underserved care settings through targeted workforce training, ethical oversight, and systematic evaluation using nursing‐sensitive outcomes.

## 1. Introduction

Artificial intelligence (AI) is a science and engineering discipline focused on enabling computer systems to emulate selected forms of human intelligent behaviour. It includes capabilities such as perception, reasoning, learning, and decision‐making and is intended to allow machines to perform defined tasks with a degree of autonomy [1–3]. Nursing first encountered AI in 1985 through expert systems designed to support clinical decision‐making [4]. Since then, rapid advances in machine learning, natural language processing (NLP), the Internet of Things, and big data analytics have expanded AI applications across health care, including nursing care for noncommunicable chronic diseases (NCDs) [5–7]. NCDs are typically long‐term, multifactorial conditions that may lead to functional impairment, disability, and reduced quality of life [8–10]. Major groups of NCDs include cardiovascular diseases, cancers, chronic respiratory diseases, and diabetes [9].

NCDs account for approximately 41 million deaths annually, representing 74% of global deaths, and place sustained demands on health systems and nursing workforces worldwide [9]. Traditional nursing care models for patients with NCDs have important limitations [9, 11, 12]. Care is often based on intermittent assessment, nurses’ clinical experience, and fixed guidelines, which can restrict personalised care and timely intervention [13, 14]. Data collection and interpretation commonly depend on manual review and episodic follow‐up, creating difficulties for long‐term monitoring, early deterioration detection, and resource allocation [15, 16]. AI technologies offer potential responses to these problems by integrating heterogeneous data and supporting risk stratification [17, 18]. They can also identify symptom patterns and generate decision‐support information for nursing teams [19–22].

In this context, AI has increasingly been positioned as an intelligent partner in the management of NCDs [23, 24]. By supporting prediction and personalisation, and by enabling prevention and proactive management, AI may improve the efficiency of chronic disease management and support patients’ quality of life. These functions are closely aligned with core nursing responsibilities, including continuity of care, symptom surveillance, self‐management support, patient education, and coordination across care settings [25, 26]. As AI tools move from technical development into clinical use, their value extends beyond algorithmic performance to integration into nursing workflows, communication processes, and person‐centred care [27–30].

Despite these opportunities, AI implementation in nursing care for NCDs remains challenging. Patients with NCDs have diverse needs across disease types, comorbidity profiles, care settings, and stages of illness [11, 31–33]. AI applications also raise concerns about patient privacy and data security [34, 35], algorithmic transparency, dataset representativeness, and accountability for AI‐supported decisions [35, 36]. Underrepresentation of nursing data and limited attention to nursing roles further constrain the development of AI tools that reflect real nursing practice [36, 37].

These issues make AI implementation a nursing leadership concern because adoption changes not only clinical information processing but also staff capability, workflow allocation, accountability, communication, and patient trust [38–40]. Implementation‐leadership research emphasises that leaders shape the introduction and sustainment of new practices in healthcare settings [41–43]. This review therefore adopts a nursing management and leadership perspective to examine the organisational conditions required for responsible and sustainable AI implementation in chronic disease care.

Accordingly, this scoping review aims to synthesise the current state of evidence on AI applications in nursing care for NCDs. Specifically, it maps (1) application scenarios, technology types, and nursing functions; (2) nursing roles and human–AI collaboration patterns; (3) care phases, outcome indicators, and implementation maturity; and (4) ethical risks and governance challenges. By mapping these dimensions, the review seeks to inform nursing management decisions on workforce preparation, workflow integration, quality assurance, and ethical governance of AI‐supported chronic disease care.

## 2. Method

The review was conducted according to the methodological framework proposed by Arksey and O’Malley [44] and was reported in accordance with the Preferred Reporting Items for Systematic Reviews and Meta‐Analyses extension for Scoping Reviews (PRISMA‐ScR) [45]. The completed PRISMA‐ScR checklist is provided as Supporting File 1. The review protocol was registered on the Open Science Framework (DOI: 10.17605/OSF.IO/PZMAF).

### 2.1. Eligibility Criteria

Eligibility criteria were developed a priori using the Population–Concept–Context (PCC) framework recommended in the JBI Manual for Evidence Synthesis [46] and are summarised in Supporting File 2. The population included adults aged 18 years or older with any type of NCDs, as well as nurses and relevant healthcare professionals who provided direct care for people with NCDs. No restrictions were applied regarding gender. The concept was the empirical application of AI techniques, including machine learning, deep learning, NLP, expert systems, and predictive algorithms, to nursing assessment, care planning, intervention, or evaluation. The context included hospital, primary care, community, home‐based, and telehealth settings.

Studies were excluded if they had no AI component, were not relevant to nursing, focused on infectious chronic diseases, were reviews, commentaries, editorials, or conference abstracts, or had no full text available after two author‐email attempts.

### 2.2. Search Strategy

The search strategy was developed through preliminary exploration of AI technologies and nursing care for NCDs. We searched MEDLINE, CINAHL, Web of Science, PsycINFO, IEEE Xplore Digital Library, ACM Digital Library, and CNKI for records published from January 1985 to June 2025. These databases were selected to provide complementary disciplinary coverage: MEDLINE for biomedical and clinical literature; CINAHL for nursing and allied health; Web of Science for multidisciplinary citation coverage; PsycINFO for behavioural and mental‐health research; IEEE Xplore and ACM Digital Library for engineering and computing studies; and CNKI for Chinese‐language evidence. This combination was intended to reduce discipline‐ and language‐related retrieval bias in a field spanning nursing, chronic disease care, and AI.

Key search terms included ‘nursing’, ‘non‐communicable disease’, ‘chronic disease’, ‘artificial intelligence’, ‘machine learning’, ‘deep learning’, ‘natural language processing’, and related synonyms and controlled vocabulary. The PubMed search string was translated by an experienced medical information specialist (author Zheng Zhang) for the other databases using equivalent controlled vocabulary and operators. The complete multidatabase search strategies are provided in Supporting File 3.

Grey literature was searched using Google Scholar to capture reports, theses, and other potentially relevant evidence not consistently indexed in bibliographic databases. Search results were sorted by the default relevance ranking, and the first 300 records were screened. This threshold followed methodological recommendations that supporting Google Scholar screening in evidence reviews should focus on approximately the first 200–300 title‐ranked records [47]. The reference lists of included studies were also hand‐searched independently by two reviewers (Shuying Miao and Chunxiang Bao).

### 2.3. Source of Evidence Selection and Data Extraction

**FIGURE 1:**
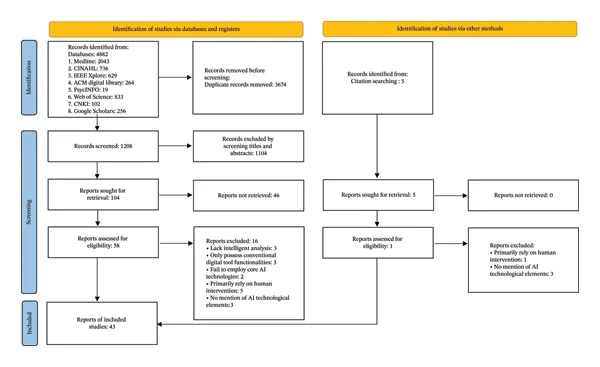
PRISMA‐ScR flowchart of study selection. Note. Inter‐reviewer agreement: Title & abstract screening: *κ* = 0.81–0.85; full‐text assessment: *κ* = 0.70–0.87. See supporting file 5.

All retrieved records were imported into EndNote X9 reference management software (Clarivate, Philadelphia, PA, USA) [48]. Duplicate records were identified using EndNote’s duplicate‐detection function and then manually verified before removal. The remaining titles and abstracts were exported to Excel for screening. Two reviewers (Shuying Miao and Chunxiang Bao) independently, and without access to each other’s decisions, completed title‐and‐abstract screening followed by full‐text assessment. Disagreements were resolved through discussion, and a third reviewer (Zheng Zhang) was consulted when consensus could not be reached. Interreviewer agreement was evaluated using Cohen’s *κ* and is reported in Supporting File 5. The study selection process is reported in the Results and illustrated in Figure 1. Data from the included studies were extracted into a structured Excel form (Supporting File 4). A full study characteristics table for all 43 included studies is provided in Supporting File 6.

### 2.4. Evidence Synthesis

A standardised data extraction form was designed, using descriptive statistics and qualitative content analysis methods [49]. According to the four evaluation questions, the extracted data were divided into two domains and frequencies, and percentages were calculated as needed.

Basic information included the author, year, title, journal or conference, country or region, type of chronic disease, sample size, research participants, research design, and research purpose. Core information included the type of AI technology, AI nursing application scenarios, nurse role, care stage, implementation obstacles, and promoting factors. These data were coded and classified.

For studies without detailed descriptions of nursing application scenarios, including nursing functions, care stages, or deployment environments, study region, research perspective, study population, research methods, and conclusions related to AI in chronic disease nursing care were extracted. Emerging themes beyond the initial framework were identified. An ‘Other’ category was added. Categories were first derived from the extracted data. They were then discussed within the review team to establish the final synthesis structure.

## 3. Results

### 3.1. Study Selection

A total of 4882 records were identified from database and grey‐literature searches. After duplicate records had been identified in EndNote and manually verified, 3674 duplicates were removed. The screening set comprised 1208 titles and abstracts; 1104 records were excluded at this stage. Fifty‐eight full‐text reports were assessed for eligibility, and 43 studies were finally included in the review (Figure 1). Interreviewer agreement ranged from 0.81 to 0.85 for title‐and‐abstract screening and from 0.70 to 0.87 for full‐text assessment, indicating substantial to near‐perfect agreement (Supporting File 5).

### 3.2. Study Characteristics

The 43 included studies were published between 2005 and 2025 and focused on AI applications in nursing care for NCDs. Research activity increased after 2020, with 34 studies (79.07%) published during this period. Mainland China and the United States each contributed 11 studies (25.58%). Other recurring locations included Taiwan, China, India, Singapore, South Korea, Australia, Italy, the United Kingdom, and multinational collaborations, each contributing two studies (4.65%). Low‐ and middle‐income countries were less represented, with India contributing two studies (4.65%) and Tunisia one study (2.33%).

Sample sizes varied substantially across the included studies. Fifteen studies (34.88%) included fewer than 100 participants, 12 studies (27.91%) enrolled 100–999 participants, and 14 studies (32.56%) involved 1000 or more participants; two studies could not be classified by sample size. Most studies focused on patient‐level data (*n* = 31, 72.09%), whereas studies targeting healthcare professionals (*n* = 5, 11.63%), textual corpora (*n* = 4, 9.30%), or patients and nurses together (*n* = 3, 6.98%) were less common.

Randomised controlled trials were uncommon, accounting for five studies (11.63%). When prospective designs (RCTs, prospective cohort studies, and other experimental studies) were considered together, they comprised only 10 studies (23.26%). The majority, therefore, relied on nonexperimental or retrospective approaches (*n* = 33, 76.74%), with retrospective cohort studies (*n* = 12, 27.91%) and observational cohort studies (*n* = 9, 20.93%) dominating the evidence base (Table 1). This pattern indicated that much of the current evidence remained exploratory and that causal evidence on AI‐supported nursing outcomes in care for NCDs was limited.

**TABLE 1:** Research overview.

Characteristic	Category	*n* (%)
Disease category	Cancer	17 (39.53%)
	Wound and ostomy‐related conditions	6 (13.95%)
	Multimorbidity	5 (11.63%)
	Cardiovascular/cerebrovascular diseases	3 (6.98%)
	Chronic respiratory diseases	3 (6.98%)
	Diabetes mellitus	3 (6.98%)
	Cognitive impairment	2 (4.65%)
	Chronic kidney disease	1 (2.33%)
	Chronic pain	1 (2.33%)
	Complex chronic diseases	1 (2.33%)
	Schizophrenia	1 (2.33%)

Country/region	China	11 (25.58%)
	United States	11 (25.58%)
	Australia	2 (4.65%)
	India	2 (4.65%)
	Italy	2 (4.65%)
	Multiple countries	2 (4.65%)
	Singapore	2 (4.65%)
	South Korea	2 (4.65%)
	Taiwan, China	2 (4.65%)
	United Kingdom	2 (4.65%)
	Germany	1 (2.33%)
	Japan	1 (2.33%)
	Norway	1 (2.33%)
	Spain	1 (2.33%)
	Tunisia	1 (2.33%)

Publication period	2020–2025	34 (79.07%)
	Before 2020	9 (20.93%)

Research participants	Patients	31 (72.09%)
	Healthcare professionals	5 (11.63%)
	Textual corpora	4 (9.30%)
	Patients and nurses	3 (6.98%)

Sample size	Small sample size (< 100)	15 (34.88%)
	Large sample size ( ≥ 1000)	14 (32.56%)
	Medium sample size (100–999)	12 (27.91%)
	Not classifiable	2 (4.65%)

Study design	Retrospective cohort study	12 (27.91%)
	Observational cohort study	9 (20.93%)
	Prospective observational study	9 (20.93%)
	Randomised controlled trial	5 (11.63%)
	Cross‐sectional study	3 (6.98%)
	Experimental study	3 (6.98%)
	Prospective cohort study	2 (4.65%)

Input data types	Unstructured	25 (58.14%)
	Structured	24 (55.81%)
	Images	5 (11.63%)

Economic considerations	Not mentioned	20 (46.51%)
	Mentioned	12 (27.91%)
	Potential cost savings	11 (25.58%)

*Note:* Percentages are based on 43 included studies. Study IDs were removed from the main table to improve readability; detailed study‐level information is provided in Supporting Table S2.

Although no formal critical appraisal was undertaken, consistent with the mapping purpose of a scoping review, the methodological profile of the evidence base was examined narratively. The predominance of nonexperimental or retrospective designs (*n* = 33, 76.74%), together with wide variation in sample size and the small proportion of RCTs (*n* = 5, 11.63%), limited causal inference. In addition, 32 studies (74.42%) remained at proof‐of‐concept or technical‐validation stages, and 14 studies (32.56%) did not report nursing or patient outcome validation. The evidence therefore provides a stronger basis for mapping application patterns and implementation issues than for establishing the clinical effectiveness of specific AI technologies.

### 3.3. AI Technology Utilisation

The included studies covered a broad range of NCDs. Cancer was the most frequently investigated condition, accounting for 17 studies (39.53%), with breast and lung cancers as the primary focus. Chronic respiratory diseases and diabetes mellitus each accounted for three studies (6.98%), and wound and ostomy‐related conditions accounted for six studies (13.95%). Mental health‐related conditions were underrepresented, with cognitive impairment examined in two studies (4.65%) and schizophrenia in one study (2.33%). Multimorbidity was examined in five studies (11.63%), but the disease combinations varied across studies (Table 1).

As presented in Table 2, the dominant AI techniques were NLP (*n* = 14, 32.56%) and risk prediction modelling (*n* = 12, 27.91%). Other techniques included computer vision (*n* = 5, 11.63%), deep learning (*n* = 4, 9.30%), supervised learning (*n* = 2, 4.65%), unsupervised learning (*n* = 2, 4.65%), neural networks (*n* = 1, 2.33%), expert systems (*n* = 1, 2.33%), intelligent robotics (*n* = 1, 2.33%), and intelligent decision‐making systems (*n* = 1, 2.33%). These patterns are relevant to nursing leadership because they indicate the types of AI literacy and technology appraisal that nurse managers need when deciding which tools can be safely introduced into nursing workflows.

**TABLE 2:** Status of AI technology implementation in nursing care (*N* = 43).

Types of Diseases	AI core technologies (*n*, %)	AI data provenance (*n*, %)	Technology readiness levels (*n*, %)	Deployment environment (*n*, %)	Ethical risks (*n*, %)	Ethics committee review (*n*, %)
All studies (*N* = 43)	Natural language processing (*n* = 14, 32.56%) Risk prediction models (*n* = 12, 27.91%) Computer vision (*n* = 5, 11.63%) Deep learning (*n* = 4, 9.30%) Supervised learning (*n* = 2, 4.65%) Unsupervised learning (*n* = 2, 4.65%) Neural networks (*n* = 1, 2.33%) Expert systems (*n* = 1, 2.33%) Intelligent robotics (*n* = 1, 2.33%) Intelligent decision‐making systems (*n* = 1, 2.33%)	Structured scales (*n* = 14, 32.56%) Electronic health records (*n* = 12, 27.91%) Web‐based data (*n* = 5, 11.63%) Specialised assessment instruments (*n* = 4, 9.30%) Clinical databases (*n* = 4, 9.30%) Patient interview data (*n* = 4, 9.30%)	Proof‐of‐concept (*n* = 16, 37.21%) Technical validation (*n* = 16, 37.21%) Clinical integration (*n* = 11, 25.58%) Autonomous decision‐making (*n* = 0)	In‐hospital settings (*n* = 20, 46.51%) Home‐based/remote monitoring (*n* = 16, 37.21%) Community settings (*n* = 3, 6.98%) Primary care applications (*n* = 2, 4.65%) Rehabilitation facilities (*n* = 1, 2.33%) Hospice wards (*n* = 1, 2.33%)	Not reported (*n* = 12, 27.91%) Uncontrollable information quality (*n* = 9, 20.93%) Data bias (*n* = 8, 18.60%) Privacy protection (*n* = 8, 18.60%) Data security (*n* = 3, 6.98%) Algorithmic bias (*n* = 2, 4.65%) Alert fatigue (*n* = 1, 2.33%)	Yes (*n* = 28, 65.12%) Not reported (*n* = 10, 23.26%) Not required (*n* = 5, 11.63%)
Cancer (*n* = 17)	Natural language processing (*n* = 9) Risk prediction (*n* = 3) Unsupervised learning (*n* = 1) Deep learning (*n* = 1) Neural networks (*n* = 1) Expert systems (*n* = 1) Intelligent decision‐making systems (*n* = 1)	Structured scales (*n* = 6) Electronic health records (*n* = 5) Web‐based data (*n* = 4) Clinical databases (*n* = 2)	Proof‐of‐concept (*n* = 10) Clinical integration (*n* = 6) Technical validation (*n* = 1)	In‐hospital settings (*n* = 9) Home‐based/remote monitoring (*n* = 5) Community settings (*n* = 1) Primary care applications (*n* = 1) Hospice wards (*n* = 1)	Uncontrollable information quality (*n* = 6) Not reported (*n* = 4) Data bias (*n* = 3) Privacy protection (*n* = 3) Alert fatigue (*n* = 1)	Yes (*n* = 9) Not reported (*n* = 5) Not required (*n* = 2)
Cardiovascular diseases (*n* = 3)	Risk prediction (*n* = 1) Supervised learning (*n* = 1) Deep learning (*n* = 1)	Structured scales (*n* = 2) Clinical databases (*n* = 1)	Technical validation (*n* = 2) Clinical integration (*n* = 1)	In‐hospital settings (*n* = 2) Home‐based/remote monitoring (*n* = 1)	Data bias (*n* = 1) Not reported (*n* = 2)	Yes (*n* = 2) Not reported (*n* = 1)
Diabetes mellitus (*n* = 3)	Risk prediction (*n* = 1) Unsupervised learning (*n* = 1) Natural language processing (*n* = 1)	Electronic health records (*n* = 2) Patient interview data (*n* = 1)	Proof‐of‐concept (*n* = 1) Technical validation (*n* = 2)	Community settings (*n* = 1) Primary care applications (*n* = 1) Home‐based/remote monitoring (*n* = 1)	Data bias (*n* = 2) Not reported (*n* = 1)	Yes (*n* = 1) Not reported (*n* = 2)
COPD (*n* = 3)	Risk prediction (*n* = 3)	Structured scales (*n* = 3)	Proof‐of‐concept (*n* = 1) Technical validation (*n* = 2)	In‐hospital settings (*n* = 1) Home‐based/remote monitoring (*n* = 2)	Uncontrollable information quality (*n* = 1) Privacy protection (*n* = 2)	Yes (*n* = 3)
Chronic pain (*n* = 1)	Natural language processing (*n* = 1)	Electronic health records (*n* = 1)	Technical validation (*n* = 1)	In‐hospital settings (*n* = 1)	Data bias (*n* = 1)	Not required (*n* = 1)
Nondiabetic CKD (*n* = 1)	Deep learning (*n* = 1)	Electronic health records (*n* = 1)	Clinical integration (*n* = 1)	Home‐based/remote monitoring (*n* = 1)	Data security (*n* = 1)	Yes (*n* = 1)
Wound and ostomy (*n* = 6)	Computer vision (*n* = 5) Risk prediction (*n* = 1)	Electronic health records (*n* = 2) Specialised assessment instruments (*n* = 3) Structured scales (*n* = 1)	Clinical integration (*n* = 3) Technical validation (*n* = 2) Proof‐of‐concept (*n* = 1)	In‐hospital settings (*n* = 5) Home‐based/remote monitoring (*n* = 1)	Algorithmic bias (*n* = 2) Privacy protection (*n* = 1) Data security (*n* = 1) Not reported (*n* = 2)	Yes (*n* = 5) Not reported (*n* = 1)
Cognitive impairment (*n* = 2)	Risk prediction (*n* = 1) Intelligent robotics (*n* = 1)	Specialised assessment instruments (*n* = 1) Patient interview data (*n* = 1)	Technical validation (*n* = 2)	In‐hospital settings (*n* = 1) Rehabilitation facilities (*n* = 1)	Not reported (*n* = 2)	Yes (*n* = 2)
Schizophrenia (*n* = 1)	Natural language processing (*n* = 1)	Web‐based data (*n* = 1)	Proof‐of‐concept (*n* = 1)	Community settings (*n* = 1)	Uncontrollable information quality (*n* = 1)	Not required (*n* = 1)
Multimorbidity (*n* = 5)	Risk prediction (*n* = 1) Supervised learning (*n* = 1) Deep learning (*n* = 1) Natural language processing (*n* = 2)	Electronic health records (*n* = 1) Structured scales (*n* = 1) Clinical databases (*n* = 1) Patient interview data (*n* = 2)	Proof‐of‐concept (*n* = 1) Technical validation (*n* = 4)	In‐hospital settings (*n* = 1) Home‐based/remote monitoring (*n* = 4)	Data bias (*n* = 1) Uncontrollable information quality (*n* = 1) Not reported (*n* = 1) Data security (*n* = 1) Privacy protection (*n* = 1)	Yes (*n* = 4) Not reported (*n* = 1)
Complex chronic disease (*n* = 1)	Risk prediction (*n* = 1)	Structured scales (*n* = 1)	Proof‐of‐concept (*n* = 1)	Home‐based/remote monitoring (*n* = 1)	Privacy protection (*n* = 1)	Yes (*n* = 1)

*Note:* Some AI technology and data‐provenance categories may not be mutually exclusive. Ethics committee review categories are mutually exclusive and sum to 43 studies.

Data sources were mainly structured assessment scales (*n* = 14, 32.56%) and electronic health records (*n* = 12, 27.91%). Web‐based data were used in five studies (11.63%), while specialised assessment instruments, clinical databases, and patient interview data were each used in four studies (9.30%). Regarding technological maturity, 16 studies (37.21%) were proof‐of‐concept investigations, 16 studies (37.21%) reached technical validation, and 11 studies (25.58%) advanced to clinical integration. No study reported a fully autonomous AI decision‐making system.

### 3.4. AI Deployment in Nursing Practice

As illustrated in Table 3, AI contributions to chronic disease nursing were concentrated in three core application modes: technology‐assisted decision support (*n* = 17, 39.53%), prevention and risk assessment (*n* = 14, 32.56%), and symptom management (*n* = 12, 27.91%). Remote patient monitoring was reported in seven studies (16.28%), health‐information support in five studies (11.63%), lifestyle intervention and early screening or diagnosis in two studies each (4.65%), and diagnostic evaluation in one study (2.33%).

**TABLE 3:** Applications of AI in nursing practice.

Types of diseases	Nursing functions (*n*, %)	Nursing role engagement (*n*, %)	Nursing phases (*n*, %)	Human–machine collaboration modes (*n*, %)	Nursing outcome validation (*n*, %)
All studies (*N* = 43)	Decision support (*n* = 17, 39.53%) Prevention and risk assessment (*n* = 14, 32.56%) Symptom management (*n* = 12, 27.91%) Remote patient monitoring (*n* = 7, 16.28%) Health‐information support (*n* = 5, 11.63%) Early screening and diagnosis (*n* = 2, 4.65%) Lifestyle intervention (*n* = 2, 4.65%) Diagnostic evaluation (*n* = 1, 2.33%)	Decision support (*n* = 17, 39.53%) Data provision (*n* = 8, 18.60%) Education (*n* = 8, 18.60%) Monitoring (*n* = 7, 16.28%) Data selection (*n* = 2, 4.65%) Model development (*n* = 1, 2.33%)	Assessment phase (*n* = 24, 55.81%) Intervention phase (*n* = 7, 16.28%) Monitoring phase (*n* = 6, 13.95%) Evaluation phase (*n* = 4, 9.30%) Planning phase (*n* = 2, 4.65%)	Technology‐assisted decision‐making (*n* = 22, 51.16%) Data entry and feedback (*n* = 7, 16.28%) Report generation and sharing (*n* = 5, 11.63%) Hybrid automation–human oversight (*n* = 5, 11.63%) Dashboard visualisation (*n* = 1, 2.33%) Mobile push notifications (*n* = 1, 2.33%) Voice assistant (*n* = 1, 2.33%) Not reported (*n* = 1, 2.33%)	Not reported (*n* = 14, 32.56%) Quality of life (*n* = 12, 27.91%) Nursing quality assessment (*n* = 8, 18.60%) Functional status (*n* = 4, 9.30%) Disease control indicators (*n* = 3, 6.98%) Hospitalisation rate (*n* = 1, 2.33%) Mortality rate (*n* = 1, 2.33%)
Cancer (*n* = 17)	Symptom management (*n* = 9) Health‐information support (*n* = 5) Prevention and risk assessment (*n* = 2) Lifestyle intervention (*n* = 1)	Decision support (*n* = 6) Education (*n* = 6) Data provision (*n* = 2) Data selection (*n* = 1) Monitoring (*n* = 1) Model development (*n* = 1)	Assessment phase (*n* = 7) Intervention phase (*n* = 4) Planning phase (*n* = 2) Education phase (*n* = 4)	Technology‐assisted decision‐making (*n* = 9) Report generation and sharing (*n* = 3) Hybrid automation–human oversight (*n* = 2) Data entry and feedback (*n* = 1) Dashboard visualisation (*n* = 1) Mobile push notifications (*n* = 1)	Quality of life (*n* = 6) Nursing quality assessment (*n* = 5) Not reported (*n* = 5) Mortality rate (*n* = 1)
Cardiovascular diseases (*n* = 3)	Remote patient monitoring (*n* = 2) Prevention and risk assessment (*n* = 1)	Decision support (*n* = 2) Monitoring (*n* = 1)	Assessment phase (*n* = 1) Intervention phase (*n* = 1) Monitoring phase (*n* = 1)	Technology‐assisted decision‐making (*n* = 2) Hybrid automation–human oversight (*n* = 1)	Functional status (*n* = 2) Not reported (*n* = 1)
Diabetes mellitus (*n* = 3)	Prevention and risk assessment (*n* = 3)	Decision support (*n* = 1) Data provision (*n* = 1) Data selection (*n* = 1)	Assessment phase (*n* = 3)	Technology‐assisted decision‐making (*n* = 2) Report generation and sharing (*n* = 1)	Hospitalisation rate (*n* = 1) Not reported (*n* = 2)
COPD (*n* = 3)	Prevention and risk assessment (*n* = 2) Remote patient monitoring (*n* = 1)	Decision support (*n* = 1) Data provision (*n* = 1) Monitoring (*n* = 1)	Monitoring phase (*n* = 2) Assessment phase (*n* = 1)	Data entry and feedback (*n* = 2) Technology‐assisted decision‐making (*n* = 1)	Quality of life (*n* = 2) Not reported (*n* = 1)
Chronic pain (*n* = 1)	Early screening and diagnosis (*n* = 1)	Decision support (*n* = 1)	Assessment phase (*n* = 1)	Technology‐assisted decision‐making (*n* = 1)	Not reported (*n* = 1)
Nondiabetic CKD (*n* = 1)	Lifestyle intervention (*n* = 1)	Education (*n* = 1)	Intervention phase (*n* = 1)	Hybrid automation–human oversight (*n* = 1)	Disease control indicators (*n* = 1)
Wound and ostomy (*n* = 6)	Prevention and risk assessment (*n* = 2) Remote patient monitoring (*n* = 2) Early screening and diagnosis (*n* = 1) Diagnostic evaluation (*n* = 1)	Decision support (*n* = 2) Data provision (*n* = 2) Monitoring (*n* = 2)	Assessment phase (*n* = 4) Monitoring phase (*n* = 2)	Data entry and feedback (*n* = 3) Technology‐assisted decision‐making (*n* = 2) Hybrid automation–human oversight (*n* = 1)	Nursing quality assessment (*n* = 3) Not reported (*n* = 2) Disease control indicators (*n* = 1)
Cognitive impairment (*n* = 2)	Prevention and risk assessment (*n* = 1) Symptom management (*n* = 1)	Decision support (*n* = 1) Monitoring (*n* = 1)	Assessment phase (*n* = 1) Intervention phase (*n* = 1)	Technology‐assisted decision‐making (*n* = 2)	Quality of life (*n* = 1) Not reported (*n* = 1)
Schizophrenia (*n* = 1)	Symptom management (*n* = 1)	Education (*n* = 1)	Assessment phase (*n* = 1)	Report generation and sharing (*n* = 1)	Quality of Life (*n* = 1)
Multimorbidity (*n* = 5)	Prevention and risk assessment (*n* = 2) Remote patient monitoring (*n* = 2) Symptom management (*n* = 1)	Data provision (*n* = 2) Decision support (*n* = 2) Monitoring (*n* = 1)	Assessment phase (*n* = 4) Monitoring phase (*n* = 1)	Technology‐assisted decision‐making (*n* = 2) Data entry and feedback (*n* = 1) Voice assistant (*n* = 1) Not reported (*n* = 1)	Quality of Life (*n* = 2) Disease control indicators (*n* = 1) Functional status (*n* = 1) Not reported (*n* = 1)
Complex chronic disease (*n* = 1)	Prevention and risk assessment (*n* = 1)	Decision support (*n* = 1)	Assessment phase (*n* = 1)	Technology‐assisted decision‐making (*n* = 1)	Functional status (*n* = 1)

*Note:* Categories summarise the main nursing functions, nurse roles, care phases, human–machine collaboration modes, and outcome validation reported in the included studies.

Most AI applications were deployed in the assessment phase of nursing care (*n* = 24, 55.81%). Fewer studies addressed the intervention phase (*n* = 7, 16.28%), monitoring phase (*n* = 6, 13.95%), evaluation phase (*n* = 4, 9.30%), or planning phase (*n* = 2, 4.65%). These findings showed that AI use in nursing care for NCDs was concentrated in assessment‐oriented functions, while applications supporting care planning, intervention delivery, and outcome evaluation remained limited.

Nurses played several roles in AI adoption. They most often acted as decision collaborators (*n* = 17, 39.53%), followed by data providers (*n* = 8, 18.60%), patient educators (*n* = 8, 18.60%), and monitoring personnel (*n* = 7, 16.28%). Roles involving data selection were reported in two studies (4.65%), and direct involvement in model development was reported in one study (2.33%). Regarding deployment settings, AI was most commonly used in hospitals (*n* = 20, 46.51%) and home‐based or remote‐monitoring settings (*n* = 16, 37.21%), whereas community settings (*n* = 3, 6.98%) and primary care (*n* = 2, 4.65%) were less frequently represented (Table 2). From a nursing leadership perspective, this concentration indicates a need to extend AI‐supported care beyond hospitals and to broaden nurses’ roles from data provision and downstream use towards AI co‐design and evaluation.

### 3.5. Outcome Validation and Ethical Governance

Outcome validation was explicitly reported in 29 studies (67.44%). Health‐related quality of life was the most frequently assessed endpoint (*n* = 12, 27.91%), followed by nursing quality assessment (*n* = 8, 18.60%), functional status (*n* = 4, 9.30%), disease control indicators (*n* = 3, 6.98%), hospitalisation rate (*n* = 1, 2.33%), and mortality rate (*n* = 1, 2.33%) (Table 3). Fourteen studies (32.56%) did not report nursing or patient outcome validation.

Ethical oversight was variably reported. Twenty‐eight studies (65.12%) reported ethics committee approval, five studies (11.63%) indicated that ethics approval was not required, and 10 studies (23.26%) did not report ethical review. Principal ethical risks included uncontrollable information quality (*n* = 9, 20.93%), data bias (*n* = 8, 18.60%), privacy protection concerns (*n* = 8, 18.60%), data security concerns (*n* = 3, 6.98%), algorithmic bias (*n* = 2, 4.65%), and alert fatigue (*n* = 1, 2.33%). Ethical risks were not reported in 12 studies (27.91%) (Table 2). For nursing leaders, these patterns highlight the need for sustained governance mechanisms, including mandatory ethics review, data security auditing, outcome monitoring frameworks, and bias assessment protocols for all AI‐integrated nursing applications.

### 3.6. Implementation Barriers and Facilitators

Through qualitative content analysis, eight overarching implementation barriers and their corresponding management‐oriented enablers were identified across the included studies (Table 4). These barriers spanned digital literacy and AI capability (*n* = 12, 27.91%), data quality and representativeness (*n* = 17, 39.53%), black‐box explainability and trust (*n* = 6, 13.95%), alert fatigue and usability (*n* = 1, 2.32%), ethical oversight and privacy (*n* = 18, 41.86%), workflow fit and workload (*n* = 12, 27.91%), interdisciplinary collaboration (*n* = 10, 23.26%), and nursing role adaptation (*n* = 9, 20.93%). The associated enablers included targeted AI literacy training, strengthened data governance, prioritisation of explainable outputs, alert optimisation, establishment of ethical review processes, workflow co‐design with frontline nurses, creation of interdisciplinary governance groups, and expansion of nursing roles in AI co‐design and evaluation. The most frequently identified barriers related to ethical governance and data quality, whereas alert fatigue was explicitly reported in only one study.

**TABLE 4:** Implementation barriers and facilitators for AI integration in nursing care for NCDs (*N* = 43).

Barrier/challenge	*n* (%)	Description/example	Facilitator
Ethical oversight and privacy	18 (41.9%)	Ten did not report an ethics review, five stated a review was not required, and the remainder identified privacy, bias, or data‐security risks.	Establish clear ethical review, privacy protection, audit trails, accountability, and bias monitoring processes.
Data quality and representativeness	17 (39.5%)	Models often relied on hospital EHRs and structured scales, while patient‐generated and community data were less represented.	Strengthen data governance, broaden data sources, and validate models across settings and patient groups.
Digital literacy and AI capability	12 (27.9%)	Nurses and patients may have limited confidence or skill in using AI‐supported tools.	Provide targeted AI literacy training, competency assessment, and escalation guidance for nurses and patients.
Workflow fit and workload	12 (27.9%)	Manual data entry, poorly integrated interfaces, and disconnected platforms may increase nursing workload.	Co‐design tools with frontline nurses, embed AI into existing workflows, and monitor workload after implementation.
Interdisciplinary collaboration	10 (23.3%)	AI implementation requires coordination among nurses, clinicians, data scientists, managers, and policy stakeholders.	Create interdisciplinary governance groups and involve nurses throughout design, validation, deployment, and evaluation.
Nursing role adaptation	9 (20.9%)	Nurses were frequently decision collaborators, data providers, educators, or monitors, but rarely model developers or validators.	Expand nursing roles in AI co‐design, local validation, implementation evaluation, and nursing‐sensitive outcome measurement.
Black‐box explainability and trust	6 (14.0%)	Deep learning and other AI models may provide limited transparency for nurses and patients.	Prioritise explainable outputs, decision rationales, and training that supports safe interpretation of model recommendations.
Alert fatigue and usability	1 (2.3%)	Excessive or poorly prioritised alerts may reduce responsiveness and usability.	Optimise alert thresholds, escalation pathways, and feedback loops to maintain clinical relevance.

*Note:* Table 4 links implementation barriers to nursing management actions, including workforce training, workflow redesign, governance, ethical oversight, and nurse involvement in AI co‐design and evaluation.

A qualitative cross‐domain comparison suggested a maturity gradient across the evidence base. Proof‐of‐concept and technical‐validation studies were predominantly assessment‐oriented, commonly used structured scales or electronic health records, and positioned nurses mainly as data providers or recipients of decision support. Clinically integrated applications more often involved remote monitoring, symptom management, patient education, and continuing nurse oversight. Across maturity levels, however, nurse participation remained concentrated downstream of model development, while applications addressing planning, intervention delivery, and outcome evaluation were uncommon. This pattern suggests that greater technical maturity was accompanied by broader workflow integration, but not yet by substantial expansion of nurses’ roles in AI co‐design, governance, or model evaluation.

## 4. Discussion

The findings are best understood through four cross‐cutting implications rather than a repetition of the descriptive frequencies: the limited maturity of the evidence base, the concentration of AI in assessment and decision support, the predominantly downstream role of nurses in AI development, and the governance requirements for responsible implementation. Together, these patterns show that AI is beginning to influence chronic disease nursing, but its integration remains uneven across diseases, care settings, nursing‐process phases, and levels of implementation maturity.

The sharp increase in studies after 2020 should be interpreted in the context of the COVID‐19 pandemic and the wider acceleration of digital health. The pandemic disrupted face‐to‐face chronic disease services and created strong demand for remote monitoring, digital triage, automated risk stratification, and data‐driven care coordination [23–25]. It also intensified the need for flexible digital models that could maintain follow‐up, symptom surveillance, and patient education when in‐person encounters were constrained [32, 34]. In this context, AI‐enabled tools were increasingly explored as part of broader digital‐health responses to COVID‐19 [50]. The post‐2020 growth in this review therefore reflects both the maturation of AI methods and a service‐level imperative to redesign chronic disease nursing for more flexible, remote, and data‐supported models of care [51–53].

The concentration of studies in China and the United States may reflect stronger investment in AI infrastructure, digital‐health policy, and large‐scale clinical data resources in these settings [54–56]. However, this distribution limits the transferability of current evidence to low‐ and middle‐income countries, primary‐care systems, and community nursing environments, where data infrastructure, workforce capacity, and implementation resources may differ substantially [57–59]. From a nursing management perspective, AI models developed in highly resourced hospital systems should not be transferred to home‐based, community‐based, or resource‐constrained NCD care without local validation, workflow adaptation, and equity assessment [60–62].

The wider literature also indicates that AI‐supported chronic disease care extends to psychiatry and cognitive impairment [63–65], older adults and geriatric nursing [66, 67], natural‐language‐processing‐supported clinical reasoning and personal health management [68, 69], and predictive or collaborative human–machine decision‐making [70–72]. These adjacent fields reinforce the need to evaluate AI not only by technical performance but also by its fit with patient populations, nursing work, and organisational decision processes.

Risk assessment emerged as one of the clearest AI application areas. In this review, risk prediction models were used to identify deterioration, complications, readmission risk, dependency, symptom clusters, and other clinically relevant states among patients with chronic conditions. In health and chronic disease contexts, AI models have been used for precision‐medicine tasks [73], lifestyle and nutrition monitoring [74, 75], smart chronic disease management [76, 77], elderly dependency assessment [78, 79], symptom and risk‐information extraction from nursing notes [80], and readmission‐risk prediction [81, 82]. For nurses, this function is valuable because it supports earlier recognition of high‐risk patients and helps prioritise monitoring, education, and follow‐up [83]. Nevertheless, risk assessment should be distinguished from demonstrated clinical benefit [84, 85]. Many included studies reported model development or technical validation, while fewer linked AI‐supported risk stratification to reduced hospitalisation, mortality, or sustained improvement in quality of life [85, 86]. Predictive accuracy alone does not ensure better nursing outcomes [86, 87]. Risk alerts and monitoring tools may also contribute to the alarm‐related workload if they are not integrated with appropriate governance and workflow safeguards [88, 89].

Clinical decision support represented a related but different application area. Whereas risk assessment identifies probability or priority, decision‐support systems aim to translate data into recommendations, alerts, care coordination prompts, or patient‐specific management suggestions [90, 91]. Examples included voice‐activated self‐monitoring [92], nurse‐driven smart‐home development [93, 94], symptom‐analysis frameworks using electronic health records [95, 96], clinical decision support for postembolisation syndrome [97], and oncology‐related NLP or machine‐learning applications [98, 99]. These applications also require attention to label bias and medical‐AI bias [60, 98], explainability and transparency [60, 100], uncertainty in human–AI and organisational decision‐making [101], and broader explainable artificial intelligence (XAI) requirements for trust and transparency [102]. The reviewed studies suggested that these systems can support nurses by organising complex information, reducing routine analytical workload, and improving consistency in symptom management and chronic disease follow‐up. At the same time, AI systems in the included studies functioned as assistive tools rather than autonomous decision‐makers. The pattern corroborates evidence on human–machine relationships and managerial human–AI collaboration [103]. It reinforces the need for human‐in‐the‐loop models in which nurses retain clinical judgement, contextual interpretation, and responsibility for patient‐centred communication [60, 93, 104].

Generative AI and large language models represent an emerging extension of decision support, but direct evidence within the included studies was limited. Potential nursing applications include summarising longitudinal records, drafting documentation, generating patient‐education materials, supporting conversational self‐management, and translating complex risk information into accessible language [27, 29, 69, 98]. These systems also pose inherent risks, including hallucinated or unverifiable content, prompt sensitivity, privacy leakage, automation bias, unequal performance across languages and cultural groups, and uncertainty about professional accountability [105–107]. The use of these systems in nursing services should therefore require human review, source‐grounded or retrieval‐grounded outputs where feasible, local validation before deployment, explicit limits on autonomous use, and continuous monitoring of safety, workload, and patient understanding [91, 108].

The limited penetration of AI into intervention design and outcome evaluation reveals an important gap in the nursing care continuum. Assessment‐oriented AI can identify risks, symptoms, and care priorities, but nursing care requires the conversion of these outputs into feasible interventions that address physiological, psychological, social, and cultural needs [109]. AI‐enabled monitoring can also support preventive nursing interventions, such as pressure injury prevention [110, 111]. This translation is complex and may explain why fewer studies examined AI‐supported care planning, intervention delivery, or postintervention evaluation [112]. Future studies should therefore move beyond model performance metrics and test whether AI‐supported nursing interventions improve patient‐centred outcomes, workflow efficiency, equity, safety, and cost‐effectiveness [40, 113].

Ethical governance and implementation barriers require greater attention. Although most studies reported ethics approval, a substantial proportion did not report ethical review or explain how privacy, data quality, algorithmic bias, transparency, and alert fatigue were managed. International governance frameworks provide a more operational structure for addressing these gaps. WHO guidance emphasises autonomy, safety, transparency, accountability, inclusiveness, and sustainability and provides specific recommendations for governing large multimodal models in health [108]. The NIST AI Risk Management Framework organises ongoing risk management around the functions Govern, Map, Measure, and Manage [114]. The European Union Artificial Intelligence Act establishes risk‐based obligations, including enhanced requirements for certain high‐risk health‐related AI systems [115].

For nursing leaders, these frameworks can be translated into concrete organisational practices: predeployment impact assessment; documentation of data provenance and representativeness; clearly assigned human‐oversight roles; privacy and cybersecurity controls; incident‐reporting pathways; periodic performance and bias audits; traceable model updates; and predefined escalation or decommissioning criteria [60, 100, 116, 117]. Governance should also include patients and frontline nurses in design, testing, and postdeployment review so that safety, equity, usability, and relational care remain visible alongside technical performance [114, 118].

Alert fatigue and alarm‐related workload remain additional risks in technology‐enabled monitoring environments [89, 119, 120]. Table 4 should therefore be interpreted as a management‐oriented synthesis linking data quality, interoperability, staff capability, workflow fit, ethical oversight, and patient acceptability to the responsibilities of nursing leaders and service managers. Recent AI‐in‐nursing and implementation studies similarly highlight the need to balance technological benefit with patient safety, workload, trust, and care‐quality outcomes [121, 122].

The findings have several implications for nursing management and leadership. AI adoption should be treated as organisational change and a governance responsibility rather than as a technical upgrade alone. Nursing leaders should actively shape technology selection, validation, implementation, and evaluation by identifying applications that address nursing‐sensitive problems and fit chronic disease workflows. They should also ensure that nurse managers and frontline nurses have sufficient AI literacy to interpret model outputs, limitations, data quality, bias, and escalation procedures.

Organisations should create structures for interdisciplinary communication and meaningful nurse involvement in AI co‐design, testing, and evaluation [123, 124]. Sustained governance is needed for data security, model monitoring, audit trails, accountability, ethical review, and equity assessment [37, 125, 126]. Implementation should be evaluated using nursing‐sensitive outcomes, patient‐reported outcomes, workload measures, safety events, and equity indicators [125, 127]. These actions can help ensure that AI augments nursing judgement and improves chronic disease care rather than introducing new risks or administrative burden.

## 5. Limitations

This review has several limitations. As a scoping review, it aimed to map the breadth and characteristics of the evidence rather than estimate pooled intervention effects. A formal critical appraisal was not undertaken; instead, the design profile, implementation maturity, and outcome‐validation patterns were synthesised narratively. The included studies were heterogeneous in disease focus, AI technology, data source, nursing role, setting, and outcome reporting, which limited direct comparison across studies. Some relevant studies may have been missed because terminology for AI and nursing applications varies widely, and grey‐literature searching is inherently difficult to reproduce. Funding sources of the included studies were not systematically charted, limiting assessment of potential sponsorship‐related influences. In addition, the rapid development of AI means that newer tools, especially generative AI and large language models, may evolve faster than the published evidence base. The findings should therefore be interpreted as a structured map of current evidence and implementation issues rather than as proof of effectiveness for specific AI technologies.

## 6. Conclusion

AI applications in nursing care for NCDs have expanded rapidly, particularly since 2020. Current evidence is concentrated in assessment, risk prediction, decision support, symptom management, and remote monitoring, while applications in intervention delivery, care planning, and outcome evaluation remain less developed. For nursing management, the central challenge is no longer whether AI can be introduced into chronic disease care but how it can be safely, equitably, and sustainably integrated into nursing workflows. This leadership framing translates the challenge into practical managerial responsibilities across the AI implementation cycle: identifying nursing‐relevant applications, appraising model evidence and workflow fit, developing workforce capability, governing data and ethical compliance, and evaluating nursing‐sensitive outcomes. Stronger nurse involvement in co‐design, workforce training, data governance, ethical oversight, and nursing‐sensitive outcome evaluation is needed to ensure that AI supports, rather than replaces, the relational and clinical judgement at the core of nursing practice.

## Author Contributions

Shuying Miao: conceptualisation, methodology, investigation, data curation, formal analysis, writing–original draft.

Zheng Zhang: methodology, investigation, data curation, writing–review and editing.

Chunxiang Bao: conceptualisation, supervision, project administration, writing–review and editing.

## Funding

This work was supported by the Discipline Construction Project Fund for Nursing Research (grant no. 2023ZYHL15) and the General Scientific Research Project of the Education Department of Zhejiang Province (Grant no. Y202353952).

## Ethics Statement

Ethics approval was not required for this scoping review, as it analysed previously published literature and did not involve human participants or identifiable personal data.

## Conflicts of Interest

The authors declare no conflicts of interest.

## Supporting Information

Additional supporting information can be found online in the Supporting Information section.

## Supporting information


**Supporting Information 1** Supporting 1. Supporting File 1 provides the completed PRISMA‐ScR checklist used to guide reporting of this review.

**Supporting Information 2** Supporting 2. Supporting File 2 presents the PCC framework and search‐term structure, including the population, concept, and context elements used to develop the eligibility criteria and search strategy.

**Supporting Information 3** Supporting 3. Supporting File 3 provides the full database and grey‐literature search strategies, including search dates, sources searched, and detailed search strings.

**Supporting Information 4** Supporting 4. Supporting File 4 provides the structured Excel data‐extraction form used during evidence charting. It contains the working extraction fields, including bibliographic details, study setting, disease category, participants, design, data type, economic considerations, and AI technology.

**Supporting Information 5** Supporting 5. Supporting File 5 presents interreviewer agreement statistics for title‐and‐abstract screening and full‐text assessment.

**Supporting Information 6** Supporting 6. Supporting File 6 presents the reader‐facing full study‐characteristics table derived from Supporting File 4. In contrast to the working extraction form, Supporting File 6 consolidates the included studies in a standardised format, while Table 1 in the main manuscript presents only high‐level aggregate characteristics to improve readability.

## Data Availability

The data that support the findings of this study are available from the corresponding author upon reasonable request.
