# Supplementary material for: Applications of Artificial Intelligence Technologies in Nursing for Noncommunicable Chronic Diseases: A Scoping Review
Source: J Nurs Manag. 2026 Aug 26;2026:5288231. doi: 10.1155/jonm/5288231 (PMC13519299; doi:10.1155/jonm/5288231)
Supplement: Supplementary file 2 [file JONM-2026-5288231-s002.docx]

**Supplementary File 2. Search Terms Based on the Population-Concept-Context (PCC) Framework for the Scoping Review**

**Population:**

- Non-Communicable Diseases
- Chronic disease
- Chronic inflammation
- Chronic pain
- Chronically infected
- Chronicity
- Chronic hepatitis
- Chronically ill
- Elderly chronic disease patients,
- multiple chronic conditions,
- Arthritis
- Arthropathy
- Osteoarthritis
- Rheumatoid arthritis
- Cancers
- tumor
- diabetes.
- Hypertension
- Cardiovascular diseases
- heart attacks
- Coronary Heart Disease, CHD
- Chronic Heart Failure
- Chronic Arrhythmia
- chronic respiratory diseases,
- Bronchial Asthma
- Interstitial Lung Disease, ILD
- Chronic Gastritis
- Inflammatory Bowel Disease (IBD)
- Liver Cirrhosis
- Chronic Pancreatitis
- Systemic Lupus Erythematosus, SLE
- Ankylosing Spondylitis
- Alzheimer’s Disease
- Parkinson’s Disease
- Multiple Sclerosis, MS
- Chronic Kidney Disease, CKD
- Chronic Pyelonephritis
- Chronic Anemia
- Chronic Leukemia
- stroke
- chronic respiratory diseases
- chronic obstructed pulmonary disease
- asthma
- hepatic diseases ,
- renal diseases,
- gastroenterological diseases.
- endocrine diseases,
- haematological diseases
- neurological diseases
- disorders diseases,
- dermatological conditions diseases,
- genetic disorders,
- trauma, mental
- disorders
- disabilities
- blindness
- deafness

**Concept:**

- Artificial intellig∗
- AI
- Artificial intelligence technology,
- Anomaly detection
- Anonymization
- Apriori algorithm
- Bayesian Networks
- clustering
- Computational linguistics,
- Computer science,
- Computer Vision,
- Conditional random field
- Convolutional neural network
- decision tree
- Deep Learning,
- Digital image processing,
- Digital health
- Data Science,
- electronic health record (EHR),
- Ensemble techniques
- Feature learning
- Feedforward neural network
- Generalized Association Rules
- Generative AI
- Generative adversarial networks (GANs)
- Gamification,
- hidden Markov models
- Information retrieval,
- Image classification,
- Image recognition,
- Information extraction
- Interface,
- Interval Data Association Rules
- k-nearest neighbor
- k-means
- Large language models (LLMs),
- Machine learning,
- Model
- mixture models
- Multi-task learning
- Multi-Relation Association Rules
- Natural language processing,
- Natural language understanding
- Neural Network
- pseudonymization
- Quantitative Association Rule
- Random Forest
- Reinforcement Learning
- Semi-supervised learning
- Sequential pattern mining
- Speech recognition,
- Similarity learning
- Supervised learning
- Support Vector Machines
- Text generation,
- Text mining
- Text classification
- unsupervised learning

**Context:**

- Nurse
- Nursing
- Chronic disease care,
- Community nursing,
- Inpatient nursing,
- Long-term nursing,
- Home-based nursing,
- Hospital nursing,
- Home nursing,
- Long-term Care Facilities,
- Outpatient nursing,
- Primary nursing providers,
- Professional nursing development
- Risk management,
- Specialist nurses,
- Nursing coordination
- Nursing leadership and management,
- Nursing practice,
- Nursing research,
- Nursing assessment,
- Nursing planning,
- Nursing implementation,
- Nursing service models,
- Nursing resource allocation,
- Nursing education,
- Nursing innovation,
- Nursing quality improvement,
- Nursing errors and prevention,
- Nursing training and assessment,
- Nursing information technology,
- Nursing data management,
- Nursing decision support systems.
