# Supplementary material for: Applications of Artificial Intelligence Technologies in Nursing for Noncommunicable Chronic Diseases: A Scoping Review
Source: J Nurs Manag. 2026 Aug 26;2026:5288231. doi: 10.1155/jonm/5288231 (PMC13519299; doi:10.1155/jonm/5288231)
Supplement: Supplementary file 3 [file JONM-2026-5288231-s003.docx]

**Supplementary File 3. Detailed Database and Grey-Literature Search Strategies**

Additional sources searched included Google Scholar, and the reference lists of included studies. Google Scholar results were screened using Google's default relevance ranking, and the first 300 records were reviewed to identify potentially eligible grey-literature reports.

MEDLINE (Ovid)

Date of Search: March 2025 (updated June 2025)

Number of results: reported in the search table below

| **Search** | **Search Terms** | **Results** |
| --- | --- | --- |
| 1 | Chronic disease.mp. or exp Chronic Disease/ | 333172 |
| 2 | Chronic inflammation.mp. | 41264 |
| 3 | Chronic pain.mp. or exp Chronic Pain/ | 68928 |
| 4 | Chronically infected.mp. | 8088 |
| 5 | Chronicity.mp. | 12297 |
| 6 | Chronic hepatitis.mp. or exp Hepatitis, Chronic/ | 57890 |
| 7 | Chronically ill.mp. or Chronic Disease/ | 298475 |
| 8 | Non-Communicable Diseases.mp. or exp Noncommunicable Diseases/ |  |
| 9 | multiple chronic conditions.mp. or exp Multiple Chronic Conditions/ | 2553 |
| 10 | Arthritis/ or exp Arthritis.mp. | 271638 |
| 11 | Arthropathy.mp. | 11451 |
| 12 | Osteoarthritis.mp. or exp Osteoarthritis/ | 127497 |
| 13 | Rheumatoid arthritis.mp. or exp Arthritis, Rheumatoid/ | 161970 |
| 14 | cancer*.mp. | 2590135 |
| 15 | tumor exp | 16564 |
| 16 | diabete*.mp. | 846768 |
| 17 | Hypertension/ or exp Hypertension.mp. | 612581 |
| 18 | Cardiovascular diseases.mp. or exp Cardiovascular Diseases/ | 254397 |
| 19 | heart attack*.mp | 7362 |
| 20 | Coronary Heart Disease.mp. or exp Coronary Disease/ | 166496 |
| 21 | Heart Failure/ or exp Chronic Heart Failure.mp. | 162825 |
| 22 | Chronic Arrhythmia.mp. or exp Arrhythmias, Cardiac/ | 69588 |
| 23 | chronic respiratory disease.mp. | 3306 |
| 24 | Bronchial Asthma.mp. or exp Asthma/ | 150634 |
| 25 | Interstitial Lung Disease.mp. or exp Lung Diseases, Interstitial/ | 24308 |
| 26 | Chronic Gastritis.mp. or Gastritis/ | 22487 |
| 27 | Inflammatory Bowel Disease.mp. or exp Inflammatory Bowel Diseases/ | 75235 |
| 28 | Liver Cirrhosis.mp. or exp Liver Cirrhosis/ | 124748 |
| 29 | Systemic Lupus Erythematosus.mp. or exp Lupus Erythematosus, Systemic/ | 85462 |
| 30 | Ankylosing Spondylitis.mp. or exp Spondylitis, Ankylosing/ | 23468 |
| 31 | Alzheimer's Disease.mp. or exp Alzheimer Disease/ | 213586 |
| 32 | Parkinson's Disease.mp. or exp Parkinson Disease/ | 144464 |
| 33 | Multiple Sclerosis.mp. or exp Multiple Sclerosis/ | 109413 |
| 34 | chronic Kidney Disease.mp. or exp Renal Insufficiency, Chronic/ | 99852 |
| 35 | Pyelonephritis/ or exp Chronic Pyelonephritis.mp. | 15293 |
| 36 | Chronic Anemia.mp. | 1344 |
| 37 | Leukemia/ or exp Chronic Leukemia.mp. | 61093 |
| 38 | Stroke/ or stroke.mp. | 421448 |
| 39 | ochronic respiratory diseases | 0 |
| 40 | Pulmonary Disease, Chronic Obstructive/ or exp chronic obstructed pulmonary disease.mp. | 55765 |
| 41 | asthma.mp. or exp Asthma/ | 212261 |
| 42 | Elderly chronic disease patients.mp. or exp "Aged, 80 and over" | 1082094 |
| 43 | Acquired Immunodeficiency Syndrome.mp. or exp Acquired Immunodeficiency Syndrome/ | 94597 |
| 44 | renal diseases.mp. | 9256 |
| 45 | gastroenterological diseases.mp. | 223 |
| 46 | endocrine diseases.mp. or exp Endocrine System Diseases/ | 1208964 |
| 47 | haematological diseases.mp. | 760 |
| 48 | neurological diseases.mp. | 21184 |
| 49 | disorders diseases.mp. | 181 |
| 50 | dermatological conditions diseases.mp. | 0 |
| 51 | genetic disorders.mp. or exp Genetic Diseases, Inborn/ | 771904 |
| 52 | trauma, mental.mp. | 146 |
| 53 | disabilities.mp. | 158672 |
| 54 | blindness.mp. or exp Blindness/ | 57922 |
| 55 | deafness.mp. or exp Deafness/ | 46427 |
| 56 | renal diseases.mp. | 9256 |
| 57 | gastroenterological diseases.mp. | 223 |
| 58 | endocrine diseases.mp. or exp Endocrine System Diseases/ | 1208964 |
| 59 | haematological diseases.mp. | 760 |
| 60 | hepatic diseases.mp. | 2230 |
| 61 | 1~60(OR) | 7119291 |
| 62 | Artificial intelligence.mp. or exp Artificial Intelligence/ | 101629 |
| 63 | Exp Artificial Intelligence/ or AI.mp. | 116932 |
| 64 | Anomaly detection.mp. | 2341 |
| 65 | Exp Data Anonymization/ or Anonymization.mp. | 796 |
| 66 | priori algorithm.mp. or exp Algorithms/ | 333757 |
| 67 | Bayesian Networks.mp. | 1936 |
| 68 | clustering.mp. or exp Cluster Analysis/ | 170602 |
| 69 | Computational linguistics.mp. | 314 |
| 70 | Computer science.mp. | 4255 |
| 71 | Computer Vision.mp. | 11662 |
| 72 | Conditional random field.mp. | 527 |
| 73 | Convolutional neural network.mp. or exp Convolutional Neural Networks/ | 25851 |
| 74 | decision tree.mp. or exp Decision Trees/ | 25614 |
| 75 | Deep Learning.mp. or exp Deep Learning/ | 89655 |
| 76 | Digital image processing.mp. or exp Image Processing, Computer-Assisted/ | 151567 |
| 77 | Digital health.mp. or exp Digital Health/ | 14222 |
| 78 | Data Science.mp. or exp Data Science/ | 4797 |
| 79 | electronic health record.mp. or exp Electronic Health Records/ | 44321 |
| 80 | Ensemble techniques.mp. | 242 |
| 81 | Feature learning.mp. | 1769 |
| 82 | Feedforward neural network.mp. or exp Feedforward Neural Networks/ | 619 |
| 83 | Generalized Association Rules.mp. | 2 |
| 84 | Generative AI.mp. | 1417 |
| 85 | Generative adversarial networks.mp. or exp Generative Adversarial Networks/ | 2473 |
| 86 | Gamification.mp. or exp Gamification/ | 2251 |
| 87 | hidden Markov models.mp. or exp Hidden Markov Models/ | 2273 |
| 88 | Information retrieval.mp. or exp "Information Storage and Retrieval"/ | 24336 |
| 89 | Image classification.mp. | 5375 |
| 90 | Image recognition.mp. | 2416 |
| 91 | Information extraction.mp. or exp "Information Storage and Retrieval"/ | 24182 |
| 92 | Interface.mp. | 274318 |
| 93 | Interval Data Association Rules.mp. | o |
| 94 | k-nearest neighbor.mp. | 4331 |
| 95 | k-means.mp. | 9562 |
| 96 | Large language models.mp. or exp Large Language Models/ | 5366 |
| 97 | Machine learning.mp. or exp Machine Learning/ | 161603 |
| 98 | mixture models.mp. | 2936 |
| 99 | model.mp. | 3174016 |
| 100 | Multi-Relation Association Rules | 0 |
| 101 | Natural language processing.mp. or exp Natural Language Processing/ | 15739 |
| 102 | Natural Language Processing/ or exp Natural language understanding.mp. | 8237 |
| 103 | Neural Networks, Computer/ or exp Neural Network.mp. | 119340 |
| 104 | pseudonymization.mp. | 105 |
| 105 | Quantitative Association Rule.mp. | 2 |
| 106 | Random Forest.mp. or exp Random Forest/ | 33498 |
| 107 | Reinforcement, Psychology/ or exp Reinforcement Learning.mp. | 25974 |
| 108 | Semi-supervised learning.mp. or Supervised Machine Learning/ | 3644 |
| 109 | Sequential pattern mining.mp. | 106 |
| 110 | Speech recognition.mp. | 6319 |
| 111 | Similarity learning.mp. | 132 |
| 112 | Supervised learning.mp. or exp Supervised Machine Learning/ | 9314 |
| 113 | Support Vector Machines.mp. or Support Vector Machine/ | 175310 |
| 114 | Text generation.mp. | 244 |
| 115 | Text mining.mp. or exp Data Mining/ | 14965 |
| 116 | Text classification.mp. | 940 |
| 117 | unsupervised learning.mp. or exp Unsupervised Machine Learning/ | 4367 |
| 118 | 62~117(OR) | 4168911 |
| 119 | nurse.mp. or exp Nurses/ | 245161 |
| 120 | Risk management.mp. or exp Risk Management/ | 34183 |
| 121 | Primary Care Nursing/ or Pediatric Nursing/ or Nursing Education Research/ | 87664 |
| 122 | Chronic disease care.mp. | 568 |
| 123 | 119~122(OR) | 8973 |
| 124 | 61 AND 118 AND 123 | 2043 |

CINAHL (EBSCO Information Services, LLC)

Date of Search: March 2025 (updated June 2025)

Number of results: reported in the search table below

| **Search** | **Search Terms** | **Results** |
| --- | --- | --- |
| 1 | Chronic disease OR MH "Chronic Disease" | 172600 |
| 2 | MH "Inflammation" OR Chronic inflammation | 56238 |
| 3 | MH "Chronic Pain" OR Chronic Pain | 53017 |
| 4 | Chronically infected | 637 |
| 5 | chronicity | 3127 |
| 6 | Chronic hepatitis OR (MH "Hepatitis, Chronic" OR MH "Hepatitis") | 13467 |
| 7 | Non-Communicable Diseases | 34,782 |
| 8 | Elderly chronic disease patient* | 447 |
| 9 | multiple chronic condition | 1636 |
| 10 | arthritis AND MH "Arthritis" | 11801 |
| 11 | MH "Arthropathy, Neurogenic" OR Arthropathy | 6604 |
| 12 | MH "Osteoarthritis" OR Osteoarthritis | 50142 |
| 13 | MH "Arthritis, Rheumatoid" OR Rheumatoid arthritis | 40274 |
| 14 | MH "Cancer Patients" AND cancer | 58116 |
| 15 | tumor | 681889 |
| 16 | diabete* OR MH "Diabetic Patients" | 273177 |
| 17 | MH "Hypertension" OR Hypertension | 144406 |
| 18 | MH "Cardiovascular Diseases" OR Cardiovascular disease* | 120777 |
| 19 | heart attacks | 75270 |
| 20 | (MH "Coronary Disease" OR MH "Heart Diseases") OR coronary heart disease | 71731 |
| 21 | MH "Heart Failure" OR Chronic Heart Failure | 54496 |
| 22 | MH "Arrhythmia" OR Chronic Arrhythmia | 14488 |
| 23 | chronic respiratory diseases | 2527 |
| 24 | MH "Asthma" OR bronchial asthma | 38355 |
| 25 | (MH "Lung Diseases, Interstitial" OR MH "Lung Diseases") OR Interstitial Lung Disease | 20221 |
| 26 | MH "Gastritis" OR Chronic Gastritis | 1909 |
| 27 | MH "Inflammatory Bowel Diseases" OR Inflammatory Bowel Disease | 15710 |
| 28 | MH "Liver Cirrhosis" OR Liver Cirrhosis | 13624 |
| 29 | MH "Pancreatitis, Chronic" OR Chronic Pancreatitis | 2058 |
| 30 | MH "Lupus Erythematosus, Systemic" OR Systemic Lupus Erythematosus | 14552 |
| 31 | (MH "Spondylitis, Ankylosing" OR MH "Spondylosis") OR ankylosing spondylitis | 6595 |
| 32 | MH "Alzheimer's Disease" OR Alzheimer’s Disease | 52948 |
| 33 | MH "Parkinson Disease" OR Parkinson’s Disease | 35755 |
| 34 | MH "Multiple Sclerosis" OR Multiple Sclerosis | 27904 |
| 35 | MH "Kidney Failure, Chronic" OR Chronic Kidney Disease | 45307 |
| 36 | Chronic Pyelonephritis OR MH "Pyelonephritis" | 1375 |
| 37 | MH "Anemia" OR Chronic Anemia | 13773 |
| 38 | chronic leukemia OR MH "Leukemia, Lymphocytic, Chronic" | 9004 |
| 39 | MH "Stroke" OR stroke | 153052 |
| 40 | ochronic respiratory diseases | 0 |
| 41 | chronic obstructed pulmonary disease OR MH "Pulmonary Disease, Chronic Obstructive" | 23213 |
| 42 | asthma OR MH "Asthma" | 51121 |
| 43 | Human Immunodeficiency Virus OR MH "Human Immunodeficiency Virus" | 137166 |
| 44 | MH "Acquired Immunodeficiency Syndrome" OR Acquired Immunodeficiency Syndrome | 20140 |
| 45 | 1~44(OR) | 2,030,936 |
| 46 | MH "Artificial Intelligence" OR Artificial intellig∗ | 16835 |
| 47 | AI | 20546 |
| 48 | Artificial intelligence technology | 1015 |
| 49 | Artificial intelligence technology OR MH "Detection Algorithms" | 1116 |
| 50 | Anonymization | 177 |
| 51 | MH "Algorithms" OR Apriori algorithm | 48486 |
| 52 | bayesian network | 1544 |
| 53 | MH "Clustering Algorithms" OR clustering | 13056 |
| 54 | MH "Linguistics" OR Computational linguistics | 5234 |
| 55 | Computer science | 831 |
| 56 | MH "Vision" OR Computer Vision | 5833 |
| 57 | Conditional random field | 152 |
| 58 | MH "Convolutional Neural Networks" OR Convolutional neural network | 2761 |
| 59 | MH "Decision Trees" OR decision tree | 5713 |
| 60 | MH "Deep Learning" OR Deep Learning | 19341 |
| 61 | (MH "Digital Imaging" OR MH "Digital Health" OR MH "Digital Technology" OR MH "Digital Media") | 12830 |
| 62 | MH "Electronic Health Records" OR electronic health records | 43187 |
| 63 | Ensemble techniques | 89 |
| 64 | Feature learning | 1829 |
| 65 | MH "Neural Networks (Computer)" OR Feedforward neural network | 4930 |
| 66 | Generalized Association Rules | 2 |
| 67 | Generative AI | 352 |
| 68 | MH "Generative Adversarial Networks" OR Generative adversarial networks | 261 |
| 69 | MH "Gamification" OR Gamification | 1108 |
| 70 | MH "Hidden Markov Models" OR hidden Markov models | 275 |
| 71 | MH "Information Retrieval" OR Information retrieval | 11688 |
| 72 | Image classification | 1197 |
| 73 | Image recognition | 486 |
| 74 | Information extraction | 3368 |
| 75 | Interface | 30064 |
| 76 | Interval Data Association Rules | 0 |
| 77 | k-nearest neighbor | 688 |
| 78 | k-means | 1388 |
| 79 | (MH "Synergy Model" OR MH "Programming Languages") OR Large language model* | 2266 |
| 80 | MH "Machine Learning" OR Machine learning | 29723 |
| 81 | mixture model* | 2474 |
| 82 | Multi-task learning | 84 |
| 83 | Multi-Relation Association Rules | 0 |
| 84 | Multi-Relation Association Rules OR MH "Natural Language Processing" | 4377 |
| 85 | Natural language understand* | 63 |
| 86 | Neural Network | 10714 |
| 87 | pseudonymization | 58 |
| 88 | Quantitative Association Rule | 1 |
| 89 | MH "Random Forest" OR Random Forest | 4292 |
| 90 | Reinforcement Learning | 571 |
| 91 | Semi-supervised learning | 104 |
| 92 | Sequential pattern mining | 28 |
| 93 | Speech recognition | 9232 |
| 94 | Similarity learning | 156 |
| 95 | Supervised learning | 1159 |
| 96 | MH "Support Vector Machine" OR Support Vector Machines | 3440 |
| 97 | Text generation | 64 |
| 98 | Text mining | 897 |
| 99 | Text classification | 324 |
| 100 | unsupervised learning | 562 |
| 101 | 46~100(OR) | 232,651 |
| 102 | Nurs* | 1,026,710 |
| 103 | Chronic disease care | 4967 |
| 104 | Community nursing OR MH "Community Health Nursing" | 38119 |
| 105 | MH "Inpatients" OR Inpatient nursing | 92096 |
| 106 | MH "Long Term Care Nursing" OR Long-term nursing | 2495 |
| 107 | Home-based nursing OR MH "Home Nursing" | 4247 |
| 108 | Hospital nursing OR MH "Hospitals" | 108464 |
| 109 | MH "Home Nursing" OR Home nursing | 62829 |
| 110 | MH "Long Term Care Nursing" OR Long-term Care Facilities | 20693 |
| 111 | MH "Outpatients" OR Outpatient nursing | 51869 |
| 112 | Primary nursing providers OR MH "Primary Nursing" | 1753 |
| 113 | Professional nursing development | 1953 |
| 114 | Risk management OR MH "Risk Management" | 32527 |
| 115 | MH "Clinical Nurse Specialists" OR Specialist nurses | 15435 |
| 116 | 102~105(OR) | 1,196,572 |
| 117 | 45 AND 101 AND 116 | 736 |
