# Supplementary material for: Applications of Artificial Intelligence Technologies in Nursing for Noncommunicable Chronic Diseases: A Scoping Review
Source: J Nurs Manag. 2026 Aug 26;2026:5288231. doi: 10.1155/jonm/5288231 (PMC13519299; doi:10.1155/jonm/5288231)
Supplement: Supplementary file 4 [file JONM-2026-5288231-s005.docx]

**Supplementary File 5. Inter-reviewer Agreement (Cohen’s Kappa) for Study Selection**

**A. Title & abstract screening (n = 1 208 records)**

| **Reviewer pair** | **A-Y B-Y** | **A-Y B-N** | **A-N B-Y** | **A-N B-N** | **Prop. agree** | **Cohen’s κ** |
| --- | --- | --- | --- | --- | --- | --- |
| Author 1 vs Author 2 | 42 | 7 | 9 | 1150 | 0.9868 | 0.8331 |
| Author 1 vs Author 3 | 41 | 8 | 10 | 1149 | 0.9851 | 0.8122 |
| Author 2 vs Author 3 | 43 | 6 | 8 | 1151 | 0.9884 | 0.8540 |

**B. Full-text screening (n = 58 reports)**

| **Reviewer pair** | **A-I B-I** | **A-I B-E** | **A-E B-I** | **A-E B-E** | **Prop. agree** | **Cohen’s κ** |
| --- | --- | --- | --- | --- | --- | --- |
| Author 1 vs Author 2 | 39 | 2 | 3 | 14 | 0.9138 | 0.7883 |
| Author 1 vs Author 3 | 38 | 3 | 4 | 13 | 0.8793 | 0.7036 |
| Author 2 vs Author 3 | 40 | 1 | 2 | 15 | 0.9483 | 0.8730 |
