# Supplementary material for: Applications of Artificial Intelligence Technologies in Nursing for Noncommunicable Chronic Diseases: A Scoping Review
Source: J Nurs Manag. 2026 Aug 26;2026:5288231. doi: 10.1155/jonm/5288231 (PMC13519299; doi:10.1155/jonm/5288231)
Supplement: Supplementary file 5 [file JONM-2026-5288231-s006.docx]

**Supplementary File 6. Full Study Characteristics of the Included Studies**

| **No.** | **Author/**  **year** | **Publication year** | **Country/**  **region** | **Disease category** | **Sample size/data source** | **Research participants** | **Study design** | **AI technology** | **Main study aim** | **Input data types** | **Economic considerations** |
| --- | --- | --- | --- | --- | --- | --- | --- | --- | --- | --- | --- |
| 1 | Bellika & Hartvigsen, 2005 | 2005 | Norway | Cancer (oncology) | 12 anonymised electronic discharge letters | Patients recently discharged from the oncology department; their electronic discharge letters | Experimental validation study comparing two IR methods (PaSent vs. TF-IDF) against human expert relevance judgements | Neural-network-based information retrieval with recurrent neural networks and context modelling; operates as an intelligent software agent (PaSent) | Develop and validate an automated, context-aware information-retrieval system that can recommend relevant medical literature to patients and health personnel after hospital discharge, thereby reducing the workload of oncological nurses. | Unstructured | Mentioned |
| 2 | Cui et al., 2024 | 2024 | China | Advanced lung cancer; common comorbidities—bone pain, cachexia, COPD, diabetes, hypertension | 949 patients | Consecutive low-income patients admitted to the hospice ward of a tertiary hospital in Wuhan | Single-center retrospective cohort; 3:1 random split into training (n = 712) and testing (n = 237) sets | (Cox proportional-hazards) + Machine learning (Decision Tree, Random Forest, Support Vector Machine, Extreme Gradient Boosting) | Compare traditional Cox regression with four machine-learning models (DT, RF, SVM, XGBoost) for predicting 60-, 90-, and 120-day mortality and identify key predictors | Structured | Not mentioned |
| 3 | Cui et al., 2024 | 2024 | China | Stroke | 523 patients | Adult acute-stroke patients admitted to a university tertiary hospital. | Prospective observational cohort (STROBE-compliant). | Random Forest (best performer among 6 ML algorithms: LR, SVM, RF, NB, GB, KNN). | Build an interpretable ML tool that early identifies patients likely to need non-home discharge, guiding resource allocation and discharge planning. | Structured; Unstructured | Potential cost savings |
| 4 | De Silva et al., 2018 | 2018 | Australia (La Trobe U & Austin Hospital) + UK (North Bristol NHS Trust) + Sweden (Luleå U-Tech) | Prostate Cancer (PCa) | 6,457 patients (from 609,960 forum posts, 22,233 unique patients) | PCa-diagnosed men who publicly discussed their treatment choice in large OSGs | Retrospective observational cohort mined from ten high-volume online support groups (OSGs) with longitudinal timeline construction | Rule/NLP + Word2Vec emotion model | Use ML & NLP to automatically extract, profile and compare patient-reported demographics, decision behaviours (Autonomous / Paternalistic / Shared), clinical factors, side-effects and emotional trajectories before and after treatment to inform personalised care and policy. | Unstructured | Mentioned |
| 5 | Dos Santos et al., 2024 | 2024 | USA | Lung cancer (non-small cell). | 1 hypothetical older adult case scenario + 22 prior validation cases. | Older hospitalized patient with post-lobectomy lung cancer. | Brief communication; iterative human-centered design with 10 refinement rounds; cross-validation against nurse-generated gold standard. | Large language model—ChatGPT (GPT-3.5 initial, GPT-4 validated). | Evaluate whether a validated prompt framework can elicit accurate, priority-ranked nursing-care plans from ChatGPT that align with gold-standard NANDA-NOC-NIC terminologies. | Unstructured | Potential cost savings |
| 6 | Gaikwad et al. (2024) | 2024 | India | Type 2 Diabetes Mellitus | 290 T2D patients with ≥1 hypoglycemic episode | Adults with Type 2 diabetes experiencing hypoglycemia | 6-month prospective cohort; 5-fold CV; supervised, unsupervised & hybrid ML comparison | Hybrid ML—Gradient Boosting, AdaBoost, Random Forest, Neural Networks, Decision Tree, SGD, SVM | Early prediction of hypoglycemia from clinical warning signs to enable timely community-nurse interventions | Structured | Potential cost savings |
| 7 | Sharon Hewner et al., 2023 | 2023 | USA (Buffalo, New York) | Diabetes (primary focus), plus hypertension, obesity, respiratory, renal, liver, cardiovascular, mental-health and substance-use disorders | 1,233 adults with diabetes (from 3,438 high-need adults) | Adult diabetic patients in an urban, low-income, diverse FQHC population | Exploratory, retrospective, nurse-guided unsupervised clustering (K-means) | Unsupervised machine learning—K-means cluster analysis enhanced by expert nursing interpretation | Rapidly segment high-need patients into clinically actionable psychosocial phenotypes using nursing knowledge + unsupervised machine learning | Structured | Not mentioned |
| 8 | Rim Jouini et al., 2022 | 2022 | Tunisia | Cardiovascular disease, stroke, kidney failure, diabetes, sleep disorders, hepatitis C (implicit through BP and ADL markers) | 3,046 elderly persons (9-year HEPESE cohort) | Hispanic elderly aged ≥65 living in the community | Retrospective, longitudinal, simulation-based predictive modeling | Hidden Markov Model (HMM) — probabilistic sequential state prediction | Predict loss of autonomy and dependency level evolution using Hidden Markov Models (HMM) and blood-pressure-guided ADL data | Structured | Not mentioned |
| 9 | Koleck et al., 2021 | 2021 | USA (Columbia University Irving Medical Center) | COPD, heart failure, type 2 diabetes mellitus (T2DM), cancer | 133,977 patients (504,395 nursing notes, 2016) | Adults ≥18 y with at least one of the four chronic conditions; 22,647-patient analytic subset | Retrospective, observational, NLP-based text-mining study | Natural Language Processing (NLP) with rule-based negation handling + multiple correspondence analysis & hierarchical clustering | Identify and compare shared vs. distinct symptom clusters among the four conditions using real-world nursing notes | Unstructured | Not mentioned |
| 10 | Lee et al., 2025 | 2025 | Taiwan | Lung adenocarcinoma | 689 (database) + 128 (clinical validation) | Adults 5–90 y with/without lung cancer (database) & 128 lung adenocarcinoma patients (validation) | Two-stage exploratory study—retrospective database modeling + prospective questionnaire validation | Supervised learning—logistic regression, neural network, Bayesian network, decision tree (best AUC = 0.893, logistic) | Build ML model relating environmental hormones to lung cancer and refine nursing risk screens | Structured | Not mentioned |
| 11 | Luo et al., 2021 | 2021 | USA (Indiana University network) | Breast cancer, colorectal cancer | 3,458 breast-cancer + 1,694 colorectal-cancer patients | Adults treated with chemotherapy (2007-2017) tracked over 0-6, 12-18, 24-30 months post-therapy | Retrospective, longitudinal; NLP + k-Modes clustering + association-rule mining | Natural language processing (UMLS MetaMap + BioWordVec embeddings) and k-Modes clustering | Discover how symptom clusters link to patient attributes (age, smoking, comorbidities, diabetes, cancer stage) | Structured; Unstructured | Mentioned |
| 12 | Rabbani et al., 2022 | 2022 | USA (Milwaukee, WI) | Type 2 diabetes + hypertension | 15 patients + 10 providers (lab); 10 patients (field pilot) | Adults with co-existing diabetes & hypertension | Iterative lab + field usability/acceptability testing of a prototype | Rule-based AI engine for reminders + Amazon Comprehend NLP for voice-to-text conversion | Build and test VoiS, a voice-activated app enabling convenient dual-biomarker tracking and clinician data sharing | Structured; Unstructured | Potential cost savings |
| 13 | Bevilacqua et al., 2023 | 2023 | Italy | Dementia (Alzheimer’s disease) | 20 older adults (10 per arm) | ≥65 y with mild-moderate dementia attending day center ≥3 mo | Pilot RCT, single-blind, 12-week intervention + 3-mo follow-up | Embedded AI in Paro (voice recognition, behavioral learning) | Evaluate if adding Paro seal-robot sessions improves QoL vs. usual care | Unstructured | Not mentioned |
| 14 | Da et al., 2024 | 2024 | China | Breast cancer | 9,504 cleaned posts (84 K raw) | Chinese breast-cancer patients & family members posting 2017-2023 | Retrospective, unsupervised text-mining of online health-community posts | Latent Dirichlet Allocation (LDA) topic modeling (Gensim) | Map evolving information & emotional needs by treatment stage to guide tailored nursing support | Unstructured | Mentioned |
| 15 | Sengupta et al., 2020 | 2020 | USA | Coronary heart disease (CHD) | 8 women, 13-week field trial | Urban women ≥50 y, post-ACS or revascularization | DSR + 13-week observational field evaluation | Rule-based decision engine + ML activity classifier (96 % accuracy) | Design and test a smartwatch + smartphone + coach dashboard system to boost walking and heart-health behaviors | Structured; Unstructured | Potential cost savings |
| 16 | Duckworth et al., 2024 | 2024 | UK | Chronic Obstructive Pulmonary Disease (COPD) | 243 COPD patients, 727 exacerbation episodes | Adults (mean age 68.8 y; 61 % male) clinically diagnosed with COPD and prescribed the myCOPD app as part of routine care. | Retrospective cohort analysis (2017-2022) + mixed-methods qualitative study (interviews & focus groups, n = 15). | Supervised machine learning – gradient-boosted decision trees (XGBoost) for binary prediction of imminent exacerbation. | Quantify the impact of user engagement patterns on the accuracy of an ML model forecasting acute COPD exacerbations ≤ 3 days in advance, and identify design strategies that balance predictive safety with patient burden. | Structured | Not mentioned |
| 17 | Jeon et al., 2023 | 2023 | South Korea | Prediabetes (pre-DM) | 31 484 nursing notes from 1 834 patients | Adult home-care patients (mean age 70.8 y; 49 % women) with any primary diagnosis. | Retrospective, single-centre, rule-based NLP development & validation vs. human-annotated gold-standard (400 notes). | Rule-based natural-language-processing classifier (exact-match & negation rules; no ML training). | Build & validate an NLP algorithm that automatically detects prediabetes-related signs/symptoms in unstructured nursing records to enable early intervention. | Unstructured | Potential cost savings |
| 18 | Kang & Kim, 2024 | 2024 | South Korea | Hepatocellular carcinoma (HCC) undergoing trans-arterial chemoembolization (TACE) | 40 RNs + 51 hospitalized patients | Oncology nurses and HCC patients with postembolization syndrome (PES) | Six-week randomized controlled trial (non-synchronized) | Supervised ML (random forest & logistic regression) for risk prediction; NLP (keyword extraction + co-occurrence matrix) for care recommendations | Evaluate whether an ML-driven CDSS improves nurses’ competence and reduces PES impact on patients’ daily life | Structured; Unstructured | Potential cost savings |
| 19 | Wang et al. 2018 | 2018 | China | Diabetic foot ulcers, venous leg ulcers, pressure ulcers, post-operative wounds, sinus/fistula wounds, and other non-healing chronic wounds. | 10 wound-care nurses (prototype trial, ≈200 cases) | Nurses from five clinical departments (Urology, Dermatology, Neurology, Endocrinology, Orthopedics). | Mixed-method user-centered design | Computer-vision-based automatic wound edge detection & area measurement; tissue composition classification (color segmentation); OCR for report & label extraction. | Replace pen-and-paper workflow with a precise, efficient, nurse-friendly mobile tool for chronic wound measurement, assessment, monitoring, and case management. | Structured; Unstructured; Images | Not mentioned |
| 20 | Dai et al., 2024 | 2024 | China | Colorectal cancer, COPD, diabetes, hypertension, heart failure | 495 patients (144 with PSH, 351 without) | Adults undergoing permanent colostomy for colorectal cancer | Retrospective case-control with 1-year follow-up, 70/30 train-test split, 5-fold CV | Random Forest (ensemble decision-tree classifier optimized using grid search) | Early identification of high-risk PSH patients and guidance of preventive nursing care | Structured | Not mentioned |
| 21 | Fritz & Dermody, 2019 | 2019 | USA & Australia | multi-morbidity (e.g., Parkinson’s, CHF, UTI, insomnia) | 10 Smart Homes (older adults ≥ 55 y) | community-dwelling older adults with ≥ 2 chronic conditions | participatory mixed-methods pilot (2015–2018); iterative nurse-annotated ground-truth creation → supervised ML training | supervised multi-algorithm ensemble (HMM, Naïve Bayes, GMM, CRF, time-series, decision trees) | embed nursing clinical knowledge into AI algorithms to detect health-state changes (falls, insomnia, treatment side-effects) and trigger timely interventions | Unstructured | Mentioned |
| 22 | Hiltunen et al., 2025 | 2025 | 7-country consortium (IT, BE, NL, PL, FI, CZ, USA) under EU Horizon 2020 | multimorbidity in home-care & nursing-home settings | ≥140 healthcare professionals (≥20 per country) | GPs, geriatricians, nurses, physiotherapists caring for ≥65-year-olds with CCC | mixed-methods pilot; pre/post surveys + hypothetical case testing with/without decision-loop (Sep 2024–Jan 2025) | supervised ML models predicting ADL/IADL decline, cognition, depression, health instability, hospitalisation & mortality | pilot-test feasibility, usability & impact of iCARE on clinical decision-making | Unstructured | Not mentioned |
| 23 | Park et al., 2024 | 2024 | USA | breast cancer (invasive) | 970 clinical notes (100 annotated) | women aged ≥18 y from Hispanic, Black, Asian, AI/AN and multiracial groups | retrospective NLP pipeline development; 70/30 split, 5-fold CV, random-search tuning | supervised text classification; TF-IDF & Doc2Vec vectors fed into Random Forest (best AUC 0.90) | automate extraction of patient-centred treatment outcomes to reveal disparities and inform equitable care strategies | Unstructured | Potential cost savings |
| 24 | Xu et al., 2024 | 2024 | China | breast cancer (invasive) | 15 real-world Q&A pairs, 3 expert nurse raters | breast-cancer nurses and patients (identified through clinical questions) | single-center, controlled experiment: GPT-4 vs RAG-GPT (random 15 Q&As, Likert evaluation) | RAG-enhanced GPT-4 (semantic retrieval + generative LLM) | test whether adding a curated local knowledge base (RAG) improves LLM response accuracy and satisfaction for breast-cancer nursing care | Unstructured | Mentioned |
| 25 | Zhang et al., 2025 | 2025 | China | mixed solid-tumor cancers receiving chemotherapy | 144 patients (72 intervention, 72 control) | adults ≥18 y undergoing chemotherapy with smartphone/WeChat access | non-randomized controlled trial; 6-month follow-up | cloud-based deep-learning predictive models for symptom detection and personalized nursing recommendations | reduce anxiety/depression and improve quality of life through AI-driven continuous nursing compared with standard care | Structured | Potential cost savings |
| 26 | Zhang et al., 2025 | 2025 | China | chronic pain (≥3 months, VAS ≥3) | 612 older adults (27.6 % with MCI) | ≥60 y chronic-pain outpatients | cross-sectional; 70/30 train-test split, SMOTETomek balancing, grid-search tuning | XGBoost (AUC 0.925) + SHAP explanations for clinical interpretability | detect early MCI risk; guide nurses to tailor preventive care | Structured | Potential cost savings |
| 27 | Macrì et al., 2025 | 2025 | Italy | chronic pain | 1 008 manually labelled notes (284 chronic / 724 non-chronic) + 42 k unlabelled notes | adult in-patients whose Italian nursing notes were retrospectively analysed | retrospective, supervised text-classification study; 80/20 stratified split, grid-search tuning, Cohen’s κ inter-rater check | XGBoost (κ = 0.94, F1 = 0.94) outperformed GBM and BERT-base-Italian; SHAP-ready for clinical explainability | benchmark and select the best ML model to auto-detect chronic-pain diagnosis in Italian clinical notes | Unstructured | Not mentioned |
| 28 | Shah-Mohammadi & Finkelstein, 2025 | 2025 | USA | cancer | 50 first-admission nursing notes from MIMIC-III | ICU cancer patients | zero-shot prompt experiment, two temperature settings (1 vs 0.3), manual expert validation | GPT-4o generative LLM in zero-shot mode | assess GPT-4o’s ability to automate symptom extraction for downstream predictive analytics | Unstructured | Not mentioned |
| 29 | Tan et al., 2024 | 2024 | China | prostate cancer | 15 common questions + 2 nursing cases, each tested 3× per model (≈ 204 generated answers) | 15 common questions / 2 nursing cases, each tested 3× per model | cross-sectional simulation; blind expert Likert scoring, non-parametric tests | zero-shot Chinese LLMs (ERNIE Bot, ChatGLM2, Spark Desk, Qwen-14B-Chat) | rank domestic LLMs (ERNIE, ChatGLM2, Spark Desk, Qwen-14B) on accuracy, comprehensiveness, readability, empathy, case analysis | Unstructured | Not mentioned |
| 30 | Kücking et al., 2024 | 2024 | Germany | chronic wounds | 210 clinicians (133 nurses, 77 physicians) | German inpatient/outpatient clinicians | simulated diagnostic experiment—six wound images, 50 % wrong AI advice, regression analysis | trained CNN classifier providing binary wound-maceration recommendations | identify clinician-level factors that increase false agreement with incorrect AI recommendations | Unstructured | Not mentioned |
| 31 | Chen et al., 2025 | 2025 | China | COPD (≥60 y) | 250 in-patients (48 % developed dysphagia) | elderly COPD in-patients | single-center, cross-sectional; logistic + C5.0 decision-tree modelling, Delong comparison | C5.0 decision-tree algorithm | identify risk factors for COPD-related dysphagia and create an easy-to-use predictive tool for preventive nursing | Structured | Not mentioned |
| 32 | Soto-Ruiz et al., 2022 | 2022 | Spain | breast cancer (post-treatment, >5 y disease-free) | 182 (91 IG + 91 CG, powered for QOL change, expandable to 192) | adult women LS-BC receiving follow-up in primary care | MRC-phase III RCT; 4-time-point assessments (pre, 3-, 6-, 9-mo) | rule-based and machine-learning algorithms that dynamically select fatigue, lymphedema, work-return etc. modules | test whether an AI-tailored digital programme improves QOL and self-efficacy vs standard nurse visits | Structured | Mentioned |
| 33 | Beck et al., 2017 | 2017 | USA | cancer (any solid or hematologic malignancy receiving chemotherapy) | 358 patients (RCT) | adult outpatients actively receiving chemotherapy | two-arm randomized controlled trial | rule-based clinical decision support system (CDSS) with guideline-driven algorithms; no machine-learning models reported. | reduce symptom burden and improve home symptom management through an integrated telehealth system. | Structured | Potential cost savings |
| 34 | Elyoseph & Levkovich, 2024 | 2024 | UK / Israel | schizophrenia | 80 AI evaluations (4 models × 10 runs × 2 vignettes) vs. 3,194 human respondents | artificial intelligence models and human respondents (clinicians\ public) | cross-sectional, case-vignette comparison | large language models (ChatGPT-3.5, ChatGPT-4, Google Bard/Bard, Anthropic Claude) – generative AI with transformer architecture. | evaluate whether LLMs can accurately predict schizophrenia prognosis and recovery outcomes compared with expert and lay perspectives. | Unstructured | Not mentioned |
| 35 | Nguyen-Truong & Fritz, 2018 | 2018 | USA | Respiratory disease, cancer, cardiovascular disease, chronic pain | Not a clinical trial; qualitative community-engaged research with Asian immigrant older adults and caregivers in Oregon | asian immigrant older adults and their family caregivers | Community-based participatory research (CBPR) and technology co-design | Supervised machine-learning models trained with clinician-in-the-loop ground truth to detect abnormal motion patterns and predict health events | Integrate minority voices into AI algorithm design to create culturally safe smart-home monitoring that reduces caregiver stress and healthcare costs while supporting aging in place. | Structured; Unstructured | Mentioned |
| 36 | Chan et al., 2021 | 2021 | Singapore | Chronic venous insufficiency; venous leg ulcers | 52 patients, 222 wound episodes, 1,621 images | adults ≥21 y with venous leg ulcers | Prospective, single-centre, cross-sectional validation study | supervised machine-learning algorithm for automated wound-boundary detection and parameter calculation. | evaluate intra- and inter-rater reliability of ML-based 3-D wound imaging versus standard manual measurement. | Unstructured; Images | Mentioned |
| 37 | Chao & Wu, 2024 | 2024 | Taiwan,China | acute MI, VT/VF, heart failure | not specified (clinical pilot data cited) | hospitalized cardiac patients (ED/ICU) and HF patients in community rehab | prospective system-development & pilot validation | resNet (8-layer CNN) + contrastive/transfer learning models for MI, VT/VF, HF detection/prediction | provide scalable, AI-driven continuous cardiac monitoring & personalized rehab to offset Taiwan’s severe nursing shortage. | Structured | Potential cost savings |
| 38 | Fong et al., 2023 | 2023 | Singapore | chronic venous insufficiency; venous leg ulcers | 82 patients, 358 wound episodes, 2,334 images | adults ≥21 y with active VLUs (68 % Chinese, 12 % Malay, 11 % Indian) | prospective cross-sectional validation | machine-learning-based 3-D wound-boundary detection algorithm embedded in TA smartphone app. | confirm TA’s reliability (ICC ≥ 0.9) for length, width, area vs manual nurse tracing in Asian skin tones. | Unstructured; Images | Not mentioned |
| 39 | dabas et al., 2025 | 2025 | Australia &Israel | pressure injuries (stage 1-3, unstageable, suspected deep-tissue injury) | 221 images from 45 aged-care residents (mean 86 ± 7 yrs) | aged-care residents with pressure injuries | cross-sectional feasibility study using annotated clinical images | classic image-processing algorithm (hsv color-space thresholding + morphological operations); unsupervised segmentation | validate an ai algorithm that automatically segments pressure injuries from routine clinical photos and calculates real-world surface area without special hardware | Unstructured; Images | Mentioned |
| 40 | Dos Santos F.C. et al., 2024 | 2024 | USA | Lung cancer (non-small cell) | 1 hypothetical older-adult case (plus 22 iterative validation cases) | hospitalized older adult with post-lobectomy lung cancer | Iterative human-centered design with expert consensus validation against gold-standard plans | Large Language Model – GPT-4 (OpenAI ChatGPT) | assess whether ChatGPT can produce high-quality, priority-ranked nursing care plans using standardized nursing terminologies (NANDA/NOC/NIC). | Unstructured | Not mentioned |
| 41 | Farmer A. et al., 2017 | 2017 | UK | chronic obstructive pulmonary disease (COPD) — moderate to very severe | 166 patients (110 EDGE, 56 usual care) | community-dwelling adults ≥40 yr with confirmed COPD (FEV1 <70% predicted, MRC dyspnea ≥2) | multicenter, open-label, RCT with 2:1 allocation (EDGE vs usual care) + embedded qualitative process evaluation | rule-based & individualized alerting algorithms (97th/3rd centile thresholds); future work plans predictive modeling. | determine efficacy of EDGE in improving COPD-specific health status (SGRQ-C) and broader clinical outcomes | Structured | Mentioned |
| 42 | Kurisu K. et al., 2025 | 2025 | Japan | all cancers (head & neck, respiratory, GI, hematological, gynecological, etc.) | 213 cancer-related suicide/attempt reports (from 620 hospitals, 2010-2023) | hospitalized adult cancer patients who died by suicide or attempted suicide while admitted | retrospective observational analysis of anonymized patient safety reports using BERTopic\ GPT-4o labeling. | BERTopic for topic modeling; GPT-4o (OpenAI API) for automated labeling of depressive symptoms, suicidal ideation, and proposed counter-measures. | Identify pre-incident risk factors and effective counter-measures for inpatient suicide prevention in oncology | Unstructured | Not mentioned |
| 43 | Liu W. et al., 2023 | 2023 | China | nondiabetic chronic kidney disease (CKD stages 1-4) | 2,060 total → 902 propensity-matched pairs (KidneyOnline vs usual care) | adults ≥18 yr with CKD (eGFR ≥15 mL/min/1.73 m², not on dialysis) | retrospective cohort with 1:1 propensity-score matching; mean follow-up 15.8 months. | deep-learning OCR for data capture\AI engine for personalized recipes, alerts, Q&A, and follow-up plans. | test whether the KidneyOnline app reduces the composite endpoint: ≥30 % eGFR fall or ESKD. | Structured; Unstructured | Mentioned |

*Note. This table consolidates all study-level characteristics from the original Table 1, providing a complete reader-facing summary of each included study. Supplementary File 4 remains the structured working data-extraction file used during evidence charting.*
